# Supplementary material for: An Analysis of the Implementation and Use of (Critical) Incident Reporting Systems ((C)IRSs) in German Hospitals: A Retrospective Cross-Sectional Study from 2017 to 2022
Source: Healthcare (Basel). 2024 Nov 27;12(23):2386. doi: 10.3390/healthcare12232386 (PMC11641434; doi:10.3390/healthcare12232386)
Supplement: Supplementary file 1 [file healthcare-12-02386-s001.zip › healthcare-3298544-supplementary.pdf]

***Table S1:*** *Participation in Cross-institutional CIRS per year.*

| | | Year | | | | | | | | | | |
|---|---|---|---|---|---|---|---|---|---|---|---|---|
| | | 2017 | | 2019 | | 2020 | | 2021 | | 2022 | | |
| | | n | Perzent (%) | n | Perzent (%) | n | Perzent (%) | n | Perzent (%) | n | Perzent (%) | p-Value |
| CIRS AINS | Participation | 141 | 5.4% | 158 | 6.1% | 133 | 5.3% | 127 | 5.2% | 118 | 4.9% | 0.443 |
| | Non-Participation | 2457 | 94.6% | 2445 | 93.9% | 2357 | 94.7% | 2328 | 94.8% | 2290 | 95.1% | |
| CIRS Berlin | Participation | 127 | 4.9% | 140 | 5.4% | 163 | 6.5% | 165 | 6.7% | 161 | 6.7% | 0.011 |
| | Non-Participation | 2471 | 95.1% | 2463 | 94.6% | 2327 | 93.5% | 2290 | 93.3% | 2247 | 93.3% | |
| Hospital CIRS | Participation | 414 | 15.9% | 583 | 22.4% | 570 | 22.9% | 563 | 22.9% | 567 | 23.5% | <0.001 |
| | Non-Participation | 2184 | 84.1% | 2020 | 77.6% | 1920 | 77.1% | 1892 | 77.1% | 1841 | 76.5% | |
| CIRS NRW | Participation | 296 | 11.4% | 337 | 12.9% | 330 | 13.3% | 320 | 13.0% | 295 | 12.3% | 0.251 |
| | Non-Participation | 2302 | 88.6% | 2266 | 87.1% | 2160 | 86.7% | 2135 | 87.0% | 2113 | 87.7% | |
| CIRS Emergency Medicine | Participation | 3 | 0.1% | 2 | 0.1% | 3 | 0.1% | 0 | 0.0% | 1 | 0.0% | 0.463 |
| | Non-Participation | 2595 | 99.9% | 2601 | 99.9% | 2487 | 99.9% | 2455 | 100.0% | 2407 | 100.0% | |
| CIRS German Surgical Society | Participation | 2 | 0.1% | 1 | 0.0% | 0 | 0.0% | 0 | 0.0% | 0 | 0.0% | 0.282 |
| | Non-Participation | 2596 | 99.9% | 2602 | 100.0% | 2490 | 100.0% | 2455 | 100.0% | 2408 | 100.0% | |
| CIRS German Pain Society | Participation | 2 | 0.1% | 2 | 0.1% | 2 | 0.1% | 2 | 0.1% | 1 | 0.0% | 0.984 |
| | Non-Participation | 2596 | 99.9% | 2601 | 99.9% | 2488 | 99.9% | 2453 | 99.9% | 2407 | 100.0% | |
| „Every mistake Counts“ | Participation | 2 | 0.1% | 5 | 0.2% | 3 | 0.1% | 2 | 0.1% | 3 | 0.1% | 0.764 |
| | Non-Participation | 2596 | 99.9% | 2598 | 99.8% | 2487 | 99.9% | 2453 | 99.9% | 2405 | 99.9% | |
| DokuPIK | Participation | 23 | 0.9% | 27 | 1.0% | 22 | 0.9% | 24 | 1.0% | 24 | 1.0% | 0.972 |
| | Non-Participation | 2575 | 99.1% | 2576 | 99.0% | 2468 | 99.1% | 2431 | 99.0% | 2384 | 99.0% | |
| CIRS Health Care | Participation | 115 | 4.4% | 133 | 5.1% | 132 | 5.3% | 138 | 5.6% | 134 | 5.6% | 0.310 |
| | Non-Participation | 2483 | 95.6% | 2470 | 94.9% | 2358 | 94.7% | 2317 | 94.4% | 2274 | 94.4% | |
| PaSIS | Participation | 32 | 1.2% | 28 | 1.1% | 29 | 1.2% | 27 | 1.1% | 26 | 1.1% | 0.982 |
| | Non-Participation | 2566 | 98.8% | 2575 | 98.9% | 2461 | 98.8% | 2428 | 98.9% | 2382 | 98.9% | |
| DGHO-CIRS | Participation | 0 | 0.0% | 0 | 0.0% | 0 | 0.0% | 0 | 0.0% | 1 | 0.0% | 0.378 |
| | Non-Participation | 2598 | 100.0% | 2603 | 100.0% | 2490 | 100.0% | 2455 | 100.0% | 2407 | 100.0% | |
| Others | Participation | 0 | 0.0% | 0 | 0.0% | 0 | 0.0% | 0 | 0.0% | 0 | 0.0% | |
| | Non-Participation | 2598 | 100.0% | 2603 | 100.0% | 2490 | 100.0% | 2455 | 100.0% | 2408 | 100.0% | |
